# Robust Privacy-Preserving Models for Cluster-Level Confounding: Recognizing Disparities in Access to Transplantation

**DOI:** 10.1007/s12561-025-09496-3

**Published:** 2025-07-07

**Authors:** Nicholas Hartman, Kevin He

**Affiliations:** 1 Department of Biostatistics, University of Michigan, 1415 Washington Heights, Ann Arbor, MI 48109 USA; 2 Kidney Epidemiology and Cost Center, University of Michigan, 1415 Washington Heights, Ann Arbor, MI 48109 USA

**Keywords:** Confounders, Correlated random effects, Empirical null, Provider profiling

## Abstract

In health services applications where the patients are clustered within common institutions or geographic regions, it is often of interest to estimate the treatment effects of the medical providers after adjusting for confounding risk factors that are related to patients’ choices of provider but beyond the providers’ control. While most existing risk-adjustment methods are only capable of controlling for patient-level confounding risk factors (e.g., age or comorbidities), there are often important cluster-level confounding variables (e.g., regional or community-level risk factors) that should be accounted for in provider evaluations. These adjustments for cluster-level confounding factors are further complicated by the limited availability of protected patient health data, the inevitable influence of unobservable confounding factors, and the presence of outlying cluster units. To address these issues, we propose a privacy-preserving model and a novel Pseudo-Bayesian inference method to robustly assess the providers’ treatment effects with adjustments for observed cluster-level confounders and corrections for overdispersion from unobserved cluster-level confounding factors. We derive theoretical connections between our proposed estimation method and the Correlated Random Effects model, uncovering several advantages in terms of estimation stability, computational efficiency, and privacy preservation. Motivated by efforts to improve equity in transplant care, we apply these methods to evaluate transplant centers while adjusting for observed geographic disparities in donor organ availability and correcting for overdispersion from unobservable confounding factors, such as the complex impact of the COVID-19 pandemic.

**Supplementary Information:**

The online version contains supplementary material available at 10.1007/s12561-025-09496-3.

## Introduction

Patient health records from the United States (U.S.) transplant registry are observed within meaningful clusters, including transplant centers, Donation Service Areas (DSAs), and broader geographic regions [1]. Typically, organ failure patients who receive treatment within the same cluster have correlated outcomes due to the common effect of the cluster on the transplantation process. For government regulators, the treatment effects of transplant centers are of particular interest, and the Centers for Medicare and Medicaid Services (CMS) use summary measures from statistical models to evaluate transplant center performance, sanctioning those deemed to provide low-quality care [2]. Considering that these sanctions may include financial penalties or suspension [3], it is essential that transplant center evaluations are based on accurate estimates of treatment quality. However, one complication in these efforts is that the inevitable influence of confounding variables can bias the treatment effect estimates [4, 5], and many of these confounding variables may be defined at the level of the clusters instead of the individual patients. Furthermore, it is almost always the case in practice that some of these confounding variables are unobservable.

In the context of transplant center evaluations, the main exposure of interest is the patient’s choice of which center to receive treatment from [6, 7]. The effect of this exposure describes the direct consequences of receiving treatment from a given center, relative to the national average treatment. Confounding arises because patients are not randomly assigned to different centers, and there is a potential for a patient’s choice of center to be associated with other cluster-level factors beyond the center’s control (e.g., environmental factors impacting the center’s local patient population). One important example of cluster-level confounding in the context of transplant care is the existence of geographic disparities in donor organ availability, which severely impact patients’ access to life-saving transplants and have been compounded by the long-lasting effects of the COVID-19 pandemic on the U.S. organ transplantation system [8]. That is, transplant centers rely on organ donations to efficiently care for their patients, and it is well documented that the rates of donation vary widely across U.S. geographic regions [9]. During the COVID-19 pandemic, certain regions were also disproportionately impacted by the increased burdens on the healthcare system, disadvantaging some organ donation networks in complex ways that are difficult to fully measure [10]. Thus, many of the centers that reside in these regions face obstacles in delivering transplants to their patients, even if they provide high-quality care. If these disparities in donor organ availability and the long-term effects of the COVID-19 pandemic are ignored, the most disadvantaged centers may be unfairly penalized under national evaluations, causing even greater inequities in access to transplantation.

In this context, direct applications of conventional models to adjust for cluster-level confounding effects, such as regional variation in donor organ availability and COVID-19 severity, would require extremely large amounts of patient-level transplant and donation records from the U.S. transplant registries. In fact, for many researchers who perform routine evaluations of transplant centers, it is prohibitively expensive and time-consuming to repeatedly request and reanalyze this amount of protected data after each quarterly update of the national databank. Therefore, many instead opt to use pre-calculated and publicly available summary statistics to evaluate U.S. transplant centers [1], despite the fact that these statistics are typically not adjusted for important cluster-level confounding factors. To overcome the limitations of this approach, one may alternatively consider implementing privacy-preserving versions of conventional adjustment models, which avoid the direct use of the patient-level registry data. However, most existing privacy-preserving models are based on decentralized optimization methods that require the individual centers to coordinate a large number of repeated calculations and communications, which is widely recognized as an unrealistic scenario [11, 12].

Other existing privacy-preserving models, such as federated learning approaches, depend on restrictive exchangeability conditions [13], which assume that all confounding factors are observed and thus are not applicable to our motivating healthcare application where many confounding factors are potentially unobservable. For example, as discussed above, the long-term impacts of the COVID-19 pandemic on organ allocation across U.S. regions are complex and difficult to fully measure with observable data [8]. Previous works have shown that unobserved confounding can cause overdispersion in the test statistics that are commonly used to detect significant treatment effects [14]. While individualized empirical null (EN) methods have been developed to estimate the severity of this overdispersion for each center and correct the test statistics accordingly [15, 16], these methods are solely designed to correct the null variances of the test statistics and are incapable of leveraging observable cluster-level confounding variables, such as regional variation in donor organ availability, to correct the conditional null means. In addition, all existing EN approaches rely on a Frequentist testing framework, which ignores variability in the confounding parameter estimates and increases the risk of falsely detecting low-quality care. Thus, new methods are needed that can estimate the effects of observed cluster-level confounding variables, incorporate these estimates into a valid inference procedure, and simultaneously account for the impact of residual confounding variables that are unobservable.

To overcome these challenges, we derive a privacy-preserving model for cluster-level confounding estimation, which only depends on public summary statistics and thus circumvents the practical obstacles in analyzing patient-level datasets. The required summary statistics are already widely used by policymakers, and the proposed estimates can be computed from a single optimization routine. By modeling functions of the summary statistics with asymptotic truncated normal densities, we develop estimators that are highly robust to outlying clusters. Finally, we propose a Pseudo-Bayesian inference method to detect underperforming transplant centers while adjusting for the observed cluster-level confounding effects and correcting for the additional impact of unobservable confounding variables on the posterior distributions. Simulations show that the proposed model accurately estimates the confounding effects, and the Pseudo-Bayesian evaluation method has a lower false-detection rate and a higher true-detection rate for significant treatment effects compared to conventional Frequentist EN approaches. We apply these methods to evaluate U.S. transplant centers, with adjustments for observed geographic disparities in donor organ availability and additional corrections for overdispersion from unobservable confounding factors, such as latent variation in pandemic-related disruptions to the organ transplantation network.

## Summary of Contributions

While our methodology is primarily motivated by transplant center evaluations and healthcare provider profiling, many of the challenges that we face in these applications are commonly observed in more general settings. Thus, our proposed models and inference procedures offer novel solutions to issues that are relevant in broader contexts. First, we will show that our approach for cluster effect estimation can achieve the same objectives as popular models for clustered data such as random effect or correlated random effect models, but it does not require individual-level data, is robust to outliers in the random effect distribution, and substantially reduces computational burden. Therefore, our proposed model is especially advantageous in settings with large and sensitive datasets and outlying cluster effects. In addition, our inference methods for identifying extreme cluster effects provide large-scale testing methods that can control for cluster-level confounding effects while simultaneously addressing overdispersion from between-cluster heterogeneity in unobserved factors. These methods are especially useful in observational studies that rely on accurate outlier detection but are subject to confounding from a wide range of sources.

## Methods

### Background and Framework

#### Notation

Throughout this paper, we introduce our proposed methods in the context of applications where patient health outcomes are of interest, and healthcare providers serve as the clustering unit under evaluation, though these methods may be applied in other settings as well. Let $i=1,\ldots ,I$ be the provider index and let $j=1,\ldots ,n_i$ be the patient index, where $n_i$ is the number of patient records within the *i*th provider. Assume that the patient-level outcome data $Y_{ij}$ are generated from a Generalized Linear Model with

1 $$\begin{aligned} g(E[Y_{ij}|\theta _{ij}^*])=\theta _{ij}^* =\mu ^*+\gamma ^*_i+\boldsymbol{X}_{ij}^{\top } \boldsymbol{\beta }+\boldsymbol{W}_i^{\top }\boldsymbol{\nu }+\alpha _i,\end{aligned}$$

where $g(\cdot )$ is the canonical link function, $\mu ^*$ is the population norm, $\gamma ^*_i$ is the treatment effect of interest that reflects the difference in quality of care between the provider and the national average, $\boldsymbol{X}_{ij}$ is a vector of patient-level variables with effects $\boldsymbol{\beta }$, $\boldsymbol{W}_i$ is a vector of observed provider-level confounding variables with effects $\boldsymbol{\nu }$, and $\alpha _i$ is an unobserved provider-level confounding term, which follows a $\text {N}(0,\sigma ^2_{\alpha })$ distribution (conditional on $\boldsymbol{X}_{i1},\ldots ,\boldsymbol{X}_{in_i}$ and $\boldsymbol{W}_i$) and is not directly related to the provider’s treatment. The assumption that $\alpha _i$ has a conditional mean of zero implies that $\alpha _i$ is independent of $\boldsymbol{X}_{i1},\ldots ,\boldsymbol{X}_{in_i}$ and $\boldsymbol{W}_i$ (we discuss in Sect. 3.2.5 how this assumption can easily be relaxed so that our method can be applied to situations where $\alpha _i$ is correlated with the observed variables). Furthermore, define the function $b(\cdot )$, with $b'(\cdot )=g^{-1}(\cdot )$, and let $a(\psi )$ be a function of the nuisance parameter, $\psi$, which is specified based on the distribution of $Y_{ij}$. We assume that $\boldsymbol{W}_i,i=1,\ldots ,I$ are independent and identically distributed and, without loss of generality, $E[\boldsymbol{W}_i]=\boldsymbol{0}$. In situations where the second condition does not hold, $\mu ^*$ can be replaced with $\mu =\mu ^*+E[\boldsymbol{W}_i^{\top }\boldsymbol{\nu }]$ and $\boldsymbol{W}_i$ can be replaced with a centered version.

While we assume that this model accurately describes the underlying data generating process for patient health outcomes, we are unable to observe these patient-level data directly in our motivating application. Thus, in this paper, we aim to develop privacy-preserving methods that only depend on summary-level information to estimate and perform inference on the underlying parameters of this model. More specifically, because $\alpha _i$ is unobserved and the patient-level transplant records are not directly accessible in our motivating application, unbiased estimation and inference cannot be obtained through conventional modeling approaches; we provide a detailed discussion of these challenges in Sect. 3.1.3. The $\gamma _i^*$ terms can be treated as either fixed parameters or random effects [17], and if $\gamma _i^*$ is assumed to be random, additional flexibility can be introduced by decomposing the overall effect of $\boldsymbol{X}_{ij}$ into a between-provider and a within-provider effect [18, 19]. This modeling framework, and its connection with our proposed methods, will be described thoroughly in later sections.

#### Sources of Variation

The different components of (1) are responsible for several sources of variation in $Y_{ij}$ across the providers:

(i) Provider treatment effects that are clinically meaningful and depart substantially from national norms ($\gamma _i^*$ values that are unusually far from zero).
(ii) Provider treatment effects that are not clinically meaningful and are similar to national norms ($\gamma _i^*$ values that follow the typical variation around zero).
(iii) Differences in the patient case-mixes of the providers (variation in $\boldsymbol{X}_{i1},\ldots ,\boldsymbol{X}_{in_i}$ across providers). For example, some transplant centers may treat high volumes of patients with certain blood types that are difficult to transplant.
(iv) Observed cluster-level confounding factors ($\boldsymbol{W}_i,i=1,\ldots ,I$), with effects $\boldsymbol{\nu }$, which impact patient outcomes and are unrelated to quality of care. For example, $\boldsymbol{W}_i$ may represent the observed geographic disparities in donor organ availability that affect transplant care but are beyond the centers’ control.
(v) Unobservable cluster-level confounding ($\alpha _i, i=1,\ldots ,I$), which impacts patient outcomes and is unrelated to quality of care. For example, $\alpha _i$ may represent a combination of many factors that are difficult to measure, such as the complex impact of the COVID-19 pandemic on the U.S. transplantation network.

The objective is to isolate the variation from source (i) above and identify outlying providers with substantial deviations from the national norms of healthcare quality. As we will describe throughout Sect. 3.2, our proposed privacy-preserving method accomplishes this through a series of interdependent steps. First, we obtain naive cluster-level test statistics (e.g., standardized Z-scores) that only account for source (iii) variation. Then, we derive a cluster-level model based on these summary statistics, which allows us to estimate the parameters $\boldsymbol{\nu }$ and $\sigma ^2_{\alpha }$ and quantify the amount of variation from sources (ii), (iv), and (v). From this information, we finally develop an improved inference procedure that accounts for this additional variation and more reliably detects outlying providers with unusual $\gamma _i^*$ values.

#### Available Summary Data

As discussed in Sect. 1, U.S. national transplant registries are maintained by a very limited number of entities, and for most policymakers, it is typically infeasible to frequently request this massive amount of protected patient-level data for routine monitoring of transplant center performance. Therefore, stakeholders within the transplantation community depend on center-level summary statistics from the Scientific Registry of Transplant Recipients (SRTR), a contractor of the U.S. Department of Health and Human Services that is responsible for reporting on the quality of providers within the transplant system [1]. These reports are publicly available and updated twice each year.

For a certain patient outcome of interest such as transplantation, death, or graft failure, the SRTR reports the number of outcome occurrences observed within each U.S. transplant center for a given time period. In addition, the SRTR computes the counterfactual number of outcome occurrences that would be expected if the center were to provide care consistent with national norms. This expected number is generated from a risk-adjustment model that controls for differences in the centers’ patient case-mixes. For example, some centers may treat many patients with high-risk conditions, which could impact the observed patient outcomes despite being unrelated to the centers’ quality of care. Conventional analyses of transplant center performance are based on indirect standardization, comparing the centers’ observed and expected outcomes.

Along with the observed and expected outcomes described above, one may obtain information on the centers’ effective sizes, which are related to the variances of the observed outcomes. Other variables related to the characteristics of the centers and their geographic regions, which could potentially serve as the $\boldsymbol{W}_i$, are also available from the public reports [1]. Using the notation described previously, we define the available summary statistics formally as $O_i=\sum _{j=1}^{n_i} Y_{ij}$, $E_i=\sum _{j=1}^{n_i} b'(\theta ^0_{ij})$, and $\tilde{n}_i=\sum _{j=1}^{n_i} b''(\theta ^0_{ij})$, where $\theta ^0_{ij}=\mu ^*+\boldsymbol{X}_{ij}^{\top } \boldsymbol{\beta }$, $O_i$ is the observed outcome, $E_i$ is the expected outcome, and $\tilde{n}_i$ is the center’s effective size. In our motivating application, we study patients’ access to transplantation across the U.S., so the main outcome of interest is the delivery of transplants to the centers’ patient populations.

It is important to note that the $E_i$ from the SRTR’s reports is only adjusted for observable patient characteristics that are known to be clinically relevant. Thus, conventional evaluations based on the SRTR’s reports account for source (iii) variation (described in Section 3.1.2) that is due to differences in patient case-mix, but they do not account for source (iv) or (v) variation caused by observed or unobserved cluster-level confounding effects. As described in earlier sections, EN methods [15, 16] have been proposed to account for both source (iii) and (v) variation, but they cannot account for source (iv) variation. These EN methods also require that $\alpha _i$ is independent of the observed variables, and the estimated confounding effect parameters are precise enough to be treated as known quantities. We propose methods to address these limitations in Sect. 3.3.2.

#### Fixed, Random, and Correlated Random Effects

The risk-adjustment models used to calculate centers’ expected outcomes can be constructed in several different ways. Conventional fixed effects (FE), random effects (RE), and correlated random effects (CRE) risk-adjustment models differ in how they specify the $\gamma _i^*$ term in (1), and these differences are directly related to the models’ properties and limitations [17]. The FE model treats $\gamma _i^*,$ $i=1,\ldots ,I$ as fixed parameters and provides unbiased estimates of $\boldsymbol{\beta }$, but it is overspecified, since $\boldsymbol{W}_i$ is also measured at the provider-level and the data do not contain sufficient information to separate the confounding effects of $\boldsymbol{W}_i$ from $\gamma ^*_i$.

An alternative modeling strategy is to treat $\gamma _i^*$ as a random quantity and $\boldsymbol{W}_i$ as a fixed covariate. This RE model circumvents the overspecification issues of the FE model [19], but it generally relies on the assumptions that $\gamma _i^*$ is independent of the fixed covariates and that $\gamma _i^*$ is generated from a common normal distribution for all *i*, which usually do not hold in provider profiling applications [17]. For example, the best-performing providers may attract certain types of patients, which violates the independence assumption, and there are almost always providers with outlying treatment effects, so it may be inappropriate to model the $\gamma _i^*$ with one common distribution.

The CRE model extends the RE model to allow for a specific correlation structure between the random effects and the fixed covariates [18]. However, the coefficient estimates may suffer from a severe lack of precision if there are outlying providers with $\gamma _i^*$ that deviate from the assumed common normal distribution [20, 21], as we show in Sect. 4. This imprecision is especially problematic for conventional evaluations based on Frequentist testing approaches, since these methods assume that the model coefficients are very precisely estimated and can be treated as known constants. While robust CRE models have been proposed to mitigate this limitation [20, 22–24], we show in Sect. 4 that the implementation of these methods is computationally infeasible for large-scale applications such as national transplant research.

The most severe limitations of all these models are that data sharing restrictions in our motivating application prevent us from fitting them directly to patient-level data, and both observed and unobserved cluster-level confounders can bias the treatment effect estimates. In Sect. 3.2 below, we overcome these challenges by proposing a modeling approach which only relies on publicly available summary statistics that are familiar to stakeholders, leverages the advantageous properties of the FE, RE, and CRE models, and seamlessly accounts for outlying transplant centers. As a first step, we obtain center-level summary statistics from the SRTR’s underspecified FE model, which ignores $\boldsymbol{W}_i$ and $\alpha _i$, but achieves unbiased and precise estimates of $\boldsymbol{\beta }$ without any distributional assumptions on $\gamma _i^*$. Then, using these naive test statistics, we derive a model to estimate $\boldsymbol{\nu }$ and allow for correlation between $\gamma _i^*$ and $\boldsymbol{X}_{i1},\ldots ,\boldsymbol{X}_{in_i}$ in the same way as the CRE model, while using asymptotic truncated normal densities to explicitly model outlying centers, correct for the impact of unobserved confounding, and provide stable estimation in the presence of extreme quality of care effects. Throughout the remainder of this paper, we refer to our proposed method as a Robust Privacy-Preserving Cluster-Level Confounding (RPP-CLC) model.

### Estimation

#### Naive Summary-Level Data

Our objective is to use the SRTR’s summary-level data to derive a likelihood function that involves the cluster-level confounding effect parameters of interest, $\boldsymbol{\nu }$ and $\sigma ^2_{\alpha }$. As a first step, we construct naive standardized Z-scores from an FE score test [16], based on the misspecified null hypotheses $H_{0i}: \gamma _i=0, i=1,\ldots ,I$, with $\gamma _i=\gamma _i^*+\alpha _i+\boldsymbol{W}_i^{\top }\boldsymbol{\nu }$:

2 $$\begin{aligned} Z_{FE,i}=\frac{O_i-E_i}{\sqrt{a(\psi )\tilde{n}_i}}. \end{aligned}$$

The limiting normal distribution of these Z-scores, which will be used in Sect. 3.2.4, is based on the asymptotic properties of the score test statistic. We discuss methods for applications with small cluster sizes in Sect. 6. We refer to these Z-scores as naive because they are only adjusted for observed patient-level factors. However, as we will see, these statistics can serve as useful intermediate data for estimating the cluster-level confounding effect parameters.

#### Model Derivations

We now derive useful formulas relating the naive Z-scores in (2) to the observed and unobserved cluster-level confounding effect parameters of interest, $\boldsymbol{\nu }$ and $\sigma ^2_{\alpha }$. We show in Appendix 1 that, for any patient-level outcome distribution in the exponential family, we can approximate the null mean and variance functions of the provider-level $Z_{FE,i}$ as

3 $$\begin{aligned} E[Z_{FE,i};\gamma ^*_i=0]\approx & \sqrt{\frac{\tilde{n}_i}{a(\psi )}}\boldsymbol{W}_i^{\top }\boldsymbol{\nu }, \end{aligned}$$

4 $$\begin{aligned} Var[Z_{FE,i};\gamma ^*_i=0]\approx & 1+\frac{\sum _{j=1}^{n_i}b'''(\theta ^0_{ij})}{\tilde{n}_i} \boldsymbol{W}_i^{\top } \boldsymbol{\nu }+\varphi \tilde{n}_i, \end{aligned}$$

where $\varphi =\sigma ^2_{\alpha }/a(\psi )$ and $\tilde{n}_i=\sum _{j=1}^{n_i} b''(\theta ^0_{ij})$ (Appendix 1). In Sect. 3.2.4, we use these results to construct an estimation algorithm for $\boldsymbol{\nu }$ and $\sigma ^2_{\alpha }$.

The formulas in (3) and (4) are written under the condition that $\gamma _i^*=0$, which implies that the center provides care exactly equal to the national expectations. Alternatively, these formulas also hold if $\gamma _i^*$ is a random quantity with $E[\gamma _i^*|\boldsymbol{W}_i,\boldsymbol{X}_{i1},\ldots ,\boldsymbol{X}_{in_i}]=0$. Because we are mainly interested in identifying outlying providers with unusual and extreme deviations from the national norm, the difference between $\gamma _i^*$ and zero under the null model is typically viewed as a consequence of fluctuations in healthcare quality that are not clinically meaningful (i.e., source (ii) from Sect. 3.1.2). If we define $U_0$ as the set of null indices corresponding to providers with $E[\gamma _i^*|\boldsymbol{W}_i,\boldsymbol{X}_{i1},\ldots ,\boldsymbol{X}_{in_i}]=0$, then $\varphi$ becomes $Var[\gamma _i^*+\alpha _i|i \in U_0,\boldsymbol{W}_i,\boldsymbol{X}_{i1},\ldots ,\boldsymbol{X}_{in_i}]/\sigma ^2_{\varepsilon }$, and all other terms in (3) and (4) are unchanged. Thus, this null model accounts for between-provider variation from a mixture of both unobserved confounding and clinically meaningless fluctuations in quality of care. Throughout this paper, we refer to centers with $\gamma ^*_i$ equal to zero (using fixed $\gamma _i^*$) or with a conditional mean of zero (using random $\gamma _i^*$) as “null centers” or “average centers” which contribute to source (ii) variation, and we refer to all other centers as “outliers” which contribute to source (i) variation. For simplicity, we introduce our methods under the framework where $\gamma _i^*$ is fixed.

Furthermore, in (1), we assumed that the distribution of $\alpha _i$, conditional on the observed covariates, is $\text {N}(0,\sigma ^2_{\alpha })$. However, the formulas in (3) and (4) also hold under the milder moment conditions that $E[\alpha _i|\boldsymbol{X}_{i1},\ldots ,\boldsymbol{X}_{in_i},\boldsymbol{W}_i]=0$ and $Var[\alpha _i|\boldsymbol{X}_{i1},\ldots ,\boldsymbol{X}_{in_i},\boldsymbol{W}_i]=\sigma ^2_{\alpha }$. Thus, for applications in which the normal distributional assumption is suspect, it is still possible to accurately estimate $\boldsymbol{\nu }$ and $\sigma ^2_{\alpha }$ using our proposed methodology. On the other hand, the normal distribution is widely used to model unobserved random variables that are outside of the providers’ control [4, 15, 25], and we argue that this is a reasonable assumption in many settings.

#### Special Cases

For certain patient-level outcome distributions, the proposed model reduces to simpler exact forms. First consider the case where $Y_{ij}|\theta ^*_{ij}$ follows a normal distribution. We show in Appendix 2 that under this assumption,

5 $$\begin{aligned} E[Z_{FE,i};\gamma _i^*=0]=\sqrt{\frac{n_i}{\sigma ^2_{\varepsilon }}}\boldsymbol{W}_i^{\top } \boldsymbol{\nu }\; \text {and}\; Var[Z_{FE,i};\gamma _i^*=0]=1+\varphi n_i, \end{aligned}$$

where $\varphi =\sigma ^2_{\alpha }/\sigma ^2_{\varepsilon }$ and $\sigma ^2_{\varepsilon }=a(\psi )$ is the nuisance parameter from (1). Therefore, for providers with $\gamma _i^*=0$, the naive Z-scores exactly follow a heteroskedastic provider-level linear model with coefficient vector $\boldsymbol{\nu }$.

Next consider the setting in which $Y_{ij}|\theta ^*_{ij}$ follows a Poisson distribution, which is the most widely assumed model for transplant outcomes in our motivating application [1]. The canonical log-link function allows us to derive exact expressions for the first two null moments:

6 $$\begin{aligned} E[Z_{FE,i};\gamma _i^*=0]= & \sqrt{\tilde{n}_i}(\exp (\boldsymbol{W}_i^{\top }\boldsymbol{\nu }+\sigma ^2_{\alpha }/2)-1), \end{aligned}$$

7 $$\begin{aligned} Var[Z_{FE,i};\gamma _i^*=0]= & \exp (\boldsymbol{W}_i^{\top }\boldsymbol{\nu }+\sigma ^2_{\alpha }/2)\nonumber \\ & \times \left[ 1+\exp (\boldsymbol{W}_i^{\top }\boldsymbol{\nu }+\sigma ^2_{\alpha }/2)(\exp (\sigma ^2_{\alpha })-1)\tilde{n}_i\right] , \end{aligned}$$

where $\tilde{n}_i=\sum _{j=1}^{n_i} \exp (\theta ^0_{ij})$ (Appendix 3). These formulas can also be easily extended to the Quasi-Poisson model by introducing $a(\psi )=\psi$ as an overdispersion parameter in (2).

#### Optimization and Robustness

If all centers were null, then the formulas in Eqs. (3)–(6) would hold for all providers, and the likelihood function for $\boldsymbol{\nu }$ and $\sigma ^2_{\alpha }$ could simply be written as a product of normal densities. With Eqs. (3)–(5), the maximum likelihood estimators (MLEs) in this case would also be ordinary least squares estimators. The problem with this approach is that there are almost always outlying or non-null providers for which these formulas do not hold, causing the “normal MLEs” to be biased. Furthermore, we do not know exactly which providers are outliers with true deviations from the national expectations, as this is the main goal of our inference procedure.

To overcome this challenge, we propose a robust version of the privacy-preserving model that recognizes outlying providers by leveraging EN estimation strategies [15, 26, 27]. First, we specify an interval $[A_{i},B_{i}]$ for each provider and assume that the $Z_{FE,i}$ scores for an outlying provider fall outside of this interval with probability one. Then, we use asymptotic truncated normal densities to model the Z-scores that fall within the null intervals. Extending the EN likelihood function from [16], we have

8 $$\begin{aligned} L(\boldsymbol{\nu },\sigma ^2_{\alpha },\pi _0)=\prod _{i \in S_0} \pi _0 \phi _i(Z_{FE,i}; \boldsymbol{\nu },\sigma ^2_{\alpha }) \prod _{i \notin S_0} \{1-\pi _0Q_i(\boldsymbol{\nu },\sigma ^2_{\alpha })\}, \end{aligned}$$

where $S_0=\{i: Z_{FE,i} \in [A_i, B_i]\}$ with cardinality $I_0$, $\phi _i$ is the normal density with mean and variance as defined in Eqs. (3)–(6), $Q_i=\int _{A_i}^{B_i} \phi _i(z) dz$, and $\pi _0$ is the null proportion. We maximize (8) with respect to $\boldsymbol{\nu }$, $\varphi$, and $\pi _0$ using numerical optimization. In our algorithm, we define initial values for the parameters by leveraging the approximate mean function in (3) and fitting a robust linear regression model. Further details, including the specification of the null intervals $[A_{i},B_{i}]$, are provided in Appendix 4.

#### Theoretical Connections With the CRE Model

In this section, we consider the scenario in which $Y_{ij}$ is generated from an underlying CRE model [17, 19], where the effects of $\boldsymbol{X}_{ij}$ are decomposed into between- and within-provider components, and we show that it has a mathematical connection with our RPP-CLC model. For simplicity, we omit $\boldsymbol{W}_i$ and $\alpha _i$ from this discussion. The CRE model allows $\gamma _i^*$ to be correlated with $\boldsymbol{X}_{ij}$ through $\boldsymbol{\bar{X}}_i$ by assuming that $\gamma _i^*=\boldsymbol{\bar{X}}_i^{\top }\boldsymbol{\xi }+\tau _i$, where $\boldsymbol{\bar{X}}_i=\sum _{j=1}^{n_i} \boldsymbol{X}_{ij}/n_i$ and $\tau _i|\boldsymbol{X}_{i1},\ldots ,\boldsymbol{X}_{in_i} \sim N(0,\sigma ^2_{\tau })$ [18, 19]. Thus, the canonical parameter of the CRE model is

9 $$\begin{aligned} \theta ^*_{ij}=\mu ^*+\tau _i+\boldsymbol{X}_{ij}^{\top }\boldsymbol{\beta }+\boldsymbol{\bar{X}}_i^{\top }\boldsymbol{\xi }, \end{aligned}$$

which can be reparameterized in terms of the between- and within-provider effects of $\boldsymbol{X}_{ij}$.

By treating $\boldsymbol{\bar{X}}_i$ as an observed provider-level confounding variable, we may estimate $\boldsymbol{\xi }$ using our proposed RPP-CLC model as an alternative to the patient-level CRE model. This implementation would first involve the collection of summary statistics from an underspecified FE model that ignores $\boldsymbol{\bar{X}}_i$ (e.g., the SRTR’s fitted model); it is well known that $\boldsymbol{\beta }$ is unbiasedly estimated in this FE model [18, 19]. Then, under the CRE model assumptions, we show in Appendix 5 that the functional form of our proposed RPP-CLC model can be expressed as $E[Z_{FE,i}|\boldsymbol{\bar{X}}_i] \approx \sqrt{\frac{\tilde{n}_i}{a(\psi )}} \boldsymbol{\bar{X}}_i^{\top }\boldsymbol{\xi }$. The exact versions of the RPP-CLC model for the Normal and Poisson distribution special cases can be derived similarly. Therefore, our RPP-CLC approach is an alternative method for fitting CRE models, with the additional advantages that it only requires provider-level summary statistics and it incorporates certain components of EN estimation methods to robustly model outlying providers. In Sect. 4, we compare our RPP-CLC model with robust versions of the CRE model, and we find that our proposed model has advantages in terms of estimation stability and computational efficiency, even if the full patient-level data are available.

We originally assumed in Model (1) that $E[\alpha _i|\boldsymbol{X}_{i1},\ldots ,\boldsymbol{X}_{in_i},\boldsymbol{W}_i]=0$, which implies that $\alpha _i$ is independent of the observed covariates. However, by adopting aspects of the CRE model, this assumption can be relaxed to allow for correlation between $\alpha _i$ and the observed covariates. That is, we may let $\alpha _i=\boldsymbol{\bar{X}}_i^{\top }\boldsymbol{\xi }+\boldsymbol{W}_i^{\top }\boldsymbol{\zeta }+\tau _i$, where $\tau _i|\boldsymbol{X}_{i1},\ldots ,\boldsymbol{X}_{in_i}, \boldsymbol{W}_i \sim \text {N}(0,\sigma ^2_{\tau })$, such that $\theta ^*_{ij}=\mu ^*+\gamma _i^*+\tau _i+\boldsymbol{X}_{ij}^{\top } \boldsymbol{\beta }+\boldsymbol{\bar{X}}_i^{\top }\boldsymbol{\xi }+\boldsymbol{W}_i^{\top }(\boldsymbol{\nu }+\boldsymbol{\zeta }),$ and $\tau _i$ serves as the unobserved quantity, satisfying all required conditions. This result is a distinction from existing EN methods, which strictly require the independence assumption for $\alpha _i$ [16].

### Inference

#### Frequentist Approach

After obtaining estimates of $\boldsymbol{\nu }$ and $\sigma ^2_{\alpha }$, our objective is to incorporate them into a valid inference procedure to identify outlying providers while adjusting for observed cluster-level confounding factors and correcting for overdispersion from heterogeneity in unobserved confounding factors. Existing approaches that account for patient-level confounding rely on a Frequentist hypothesis testing framework, where it is assumed that $\boldsymbol{\beta }$ can be estimated very precisely and treated as a known quantity [16, 28]. The justification for this assumption is that $\widehat{\boldsymbol{\beta }}$ is typically derived from millions of patient records included in the national registries.

In contrast, the precision of $\widehat{\boldsymbol{\nu }}$ and $\widehat{\sigma }^2_{\alpha }$ increases with the number of unique providers instead of the number of patient records. Thus, even with massive patient-level datasets, the Frequentist testing approach is only appropriate for handling cluster-level confounding when a very large number of providers is under evaluation. If one were to proceed with this approach, a corrected version of the Z-scores, $Z_{FE,i}^*$, could be computed as

10 $$\begin{aligned} Z^*_{FE,i}=\frac{Z_{FE,i}-\widehat{E}[Z_{FE,i};\gamma _i^*=0]}{\sqrt{\widehat{Var}[Z_{FE,i};\gamma ^*_i=0]}}, \end{aligned}$$

where $\widehat{E}[Z_{FE,i};\gamma _i^*=0]$ and $\widehat{Var}[Z_{FE,i};\gamma _i^*=0]$ are obtained by plugging $\widehat{\boldsymbol{\nu }}$ and $\widehat{\sigma }^2_{\alpha }$ into the conditional moments from Eqs. (3)–(6). One may test whether a provider’s care deviates from the national norm by comparing $Z_{FE,i}^*$ to a quantile of the N(0,1) distribution.

In practice, flagging methods are typically applied at a national level to evaluate all providers, and multiple testing issues can inflate the overall false-flagging rates. Therefore, one may consider the use of standard multiple testing corrections, which can easily be applied along with our proposed flagging methods by adjusting the significance thresholds. However, in the context of healthcare provider evaluations, the negative consequences of failing to detect low-quality care are often viewed as more severe than the consequences of falsely flagging a provider. This is because Type II errors allow low-quality providers to continue providing poor care to patients without consequence. Thus, in some cases, inflated false-flagging rates due to multiple testing may be more tolerable than any reduction in power.

#### Pseudo-Bayesian Approach

In many applications, the number of providers under evaluation is modest, and there may be non-negligible variability in the confounding effect estimates that should be accounted for. For example, in our motivating application, there are only 256 U.S. kidney transplant programs, which we argue is not enough to estimate the confounding parameters with near-zero sampling variability. Furthermore, clinicians prefer to interpret quality of care using indirectly standardized ratios of observed and expected outcomes, but Frequentist EN methods cannot account for the impact of cluster-level confounding without first converting these ratios to Z-score statistics, which are substantially less clinically meaningful. In addition, very small centers can have unstable and highly extreme measure values, making the inference results less reliable [29]. To avoid the inappropriate use of Frequentist testing methods under these scenarios, we develop a Pseudo-Bayesian inference procedure that accounts for the uncertainty in the cluster-level confounding parameter estimates and can be interpreted on the measure ratio scale. To substantially simplify the analytic formulas in our derived posterior distributions, we focus on the uncertainty in $\widehat{\boldsymbol{\nu }}$ and treat $\sigma ^2_{\alpha }$ as a known nuisance parameter. Here, we use the term “Pseudo-Bayesian” to emphasize that our posterior distributions are approximated based on this approach, though in general fully Bayesian methods can be implemented. We find empirically in Sect. 4 that the variability in $\widehat{\boldsymbol{\nu }}$ tends to be much more consequential than the variability in $\sigma ^2_{\alpha }$, and this Pseudo-Bayesian approximation has very desirable statistical properties.

Under the Pseudo-Bayesian framework, $\boldsymbol{\nu }$ is a random quantity, and we propose a multivariate normal prior distribution for $\boldsymbol{\nu }$, $\text {MVN}(\boldsymbol{0},\boldsymbol{\Sigma _{\text {prior}}})$. Asymptotically, $\widehat{\boldsymbol{\nu }} \sim \text {MVN}(\boldsymbol{\nu },\boldsymbol{\Sigma _{\widehat{\nu }}})$, and we show in Appendix 6 that

$$\boldsymbol{\Sigma _{\widehat{\nu }}} \approx (\boldsymbol{W^*_{\text {null}}}^{\top }\boldsymbol{W^*_{\text {null}}})^{-1}\boldsymbol{W^*_{\text {null}}}^{\top }\boldsymbol{\Omega W^*_{\text {null}}}(\boldsymbol{W^*_{\text {null}}}^{\top }\boldsymbol{W^*_{\text {null}}})^{-1},$$

where $\boldsymbol{W^*_{\text {null}}}$ is an $I_0$ by *P* matrix of null providers with rows $\sqrt{\frac{\tilde{n}_i}{a(\psi )}}\boldsymbol{W}_i$ and $\boldsymbol{\Omega }$ is an $I_0$ by $I_0$ diagonal matrix with diagonal elements $Var[Z_{FE,i}|\boldsymbol{W}_i,\gamma _i^*=0]$ from Eq. (4) or (6). Asymptotically, the posterior distribution of $\boldsymbol{\nu }$ is $\text {MVN}(\boldsymbol{m_{\text {post}}},\boldsymbol{\Sigma _{\text {post}}})$, where $\boldsymbol{m_{\text {post}}}=\boldsymbol{\Sigma _{\text {prior}}}\left( \boldsymbol{\Sigma _{\text {prior}}}+\boldsymbol{\Sigma _{\widehat{\nu }}}\right) ^{-1}\widehat{\boldsymbol{\nu }}$ and $\boldsymbol{\Sigma _{\text {post}}}=\left( \boldsymbol{\Sigma }^{-1}_{\text {prior}}+\boldsymbol{\Sigma }^{-1}_{\widehat{\boldsymbol{\nu }}} \right) ^{-1}$. We then incorporate the unobserved quantity, $\alpha _i$, by deriving an approximate $\text {Lognormal}(\boldsymbol{W}_i^{\top }\boldsymbol{m_{\text {post}}},\boldsymbol{W}_i^{\top } \boldsymbol{\Sigma _{\text {post}} W}_i+\sigma ^2_{\alpha })$ posterior distribution for a useful random variable, $\Lambda _i=\exp (\boldsymbol{W}_i^{\top }\boldsymbol{\nu }+\alpha _i)$, describing the total (observed and unobserved) cluster-level confounding effects.

Let $R_i$ denote a random variable that corresponds to the true value of the naive healthcare quality measure ratio, which is not adjusted for $\boldsymbol{W}_i$ or the influence of $\alpha _i$. For measures of access to transplantation, the SRTR uses a Gamma-Poisson prior-likelihood framework [4, 29] to specify the posterior distribution of $R_i$ (denoted as $f_{R_i|O_i,E_i}(r_i)$) as Gamma$(O_i+2,E_i+2)$. We extend this framework to derive the following approximate posterior distribution for a corrected random variable of healthcare quality, $R_i^*$, which takes into account the cluster-level confounding effects:

11 $$\begin{aligned} f_{R^*_i|O_i,E_i}(r^*_i)=\int f_{R_i^*|O_i,E_i,\Lambda _i}(r^*_i)f_{\Lambda _i|O_i,E_i}(\lambda _i) \, \mathrm{d}\lambda _i,\end{aligned}$$

where $f_{R_i^*|O_i,E_i,\Lambda _i}(r^*_i)$ is the Gamma$(O_i+2,E_i\lambda _i+2)$ density function and $f_{\Lambda _i|O_i,E_i}(\lambda _i)$ is the approximate Lognormal posterior density function for $\Lambda _i$ defined above (Appendix 7). Outlying providers are flagged based on the posterior credible intervals.

## Simulations

### Estimation

We assessed the accuracy of our estimation procedure through numerical evaluations. While $\gamma _i^*$ is the main parameter of interest, accurate estimation of $\boldsymbol{\nu }$ and $\sigma ^2$ is a crucial first step that allows us to account for cluster-level confounding in our evaluations and accurately identify clusters with extreme $\gamma _i^*$. In our motivating application of United States transplant center evaluation, there are approximately 200 transplant centers with patient populations of varying sizes. Therefore, to emulate this scenario, we simulated the outcome data from a Poisson model for 200 clusters, with $n_i$ varying from 50 to 450. A single observed cluster-level confounding variable, $W_i$, was generated from a $\text {N}(0,1)$ distribution, and the unobserved quantity, $\alpha _i$, was generated from a $\text {N}(0,0.1)$ distribution. The effect of $W_i$ was set as $\nu =0.25$. For some proportion of providers, we set $\gamma ^*_i=0$. Then, for the remaining providers, we set $\gamma ^*_i=c+0.5W_i$, where *c* is a non-zero constant. We varied the proportion of providers with $\gamma ^*_i \ne 0$ (i.e., the “outlier proportion”) and the magnitude of *c* for the outlying providers. The population norm was set as $\mu =-6$. A single patient-level risk factor $X_{ij}$ with effect $\beta =1$ was simulated from a Normal($-$0.4,0.5) distribution, and the patient-level outcomes $Y_{ij}$ were simulated from Poisson($\exp (\theta _{ij}^*$)) distributions, where

12 $$\begin{aligned} \theta _{ij}^*=\mu ^*+\gamma ^*_i+\beta X_{ij}+\nu W_i+\alpha _i. \end{aligned}$$

The observed and expected summary statistics that emulate the available data in our motivating application were calculated as $O_i=\sum _{j=1}^{n_i} Y_{ij}$ and $E_i=\sum _{j=1}^{n_i} \exp (\mu ^*+\beta X_{ij}),$ respectively. For each simulation setting, we computed $Z_{FE,i}$ values from (2) and estimated $\nu$ and $\sigma _{\alpha }^2$ using both the normal MLE and the proposed RPP-CLC methods described in Sect. 3.2.

**Fig. 1:**
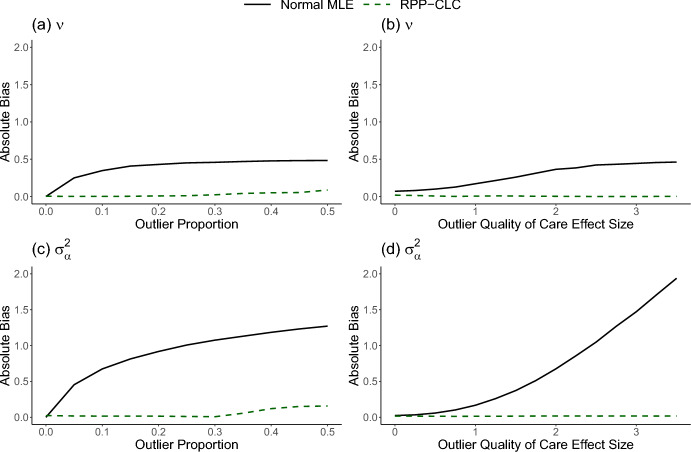
Absolute value of the bias in the cluster-level confounding effect estimates, $\widehat{\nu }$ and $\widehat{\sigma }_{\alpha }^2$, estimated via the normal Maximum Likelihood Estimator (MLE) or the proposed Robust Privacy-Preserving model for Cluster-Level Confounding (RPP-CLC model). **a**, **c** The outlier quality of care effect size is fixed at two, and the outlier proportion is varied. **b**, **d** The outlier proportion is fixed at 0.1, and the outlier quality of care effect size is varied. Results are based on 1000 iterations

In Fig. 1, we observed that both methods produced nearly unbiased estimates of $\nu$ and $\sigma ^2_{\alpha }$ when there were no outliers. However, as expected based on the arguments in Sect. 3.2, the estimates from the normal MLE approach became much more biased than those from the RPP-CLC method as the outlier proportion and the outlier effect size increased. The RPP-CLC approach was highly robust to the outlying providers, and the estimates remained close to unbiased across all settings.

### Comparisons with the CRE Model

In Sect. 3.1, we showed that our RPP-CLC model can estimate between-provider and within-provider covariate effects like the CRE model. As described in Sect. 3.1.4, this connection is important because the CRE model is often considered as a method to circumvent violations in the RE assumptions that are typical in provider profiling, but the practical utility of the CRE model is limited in this setting because it relies on sensitive patient-level data and robust versions can be very computationally expensive. To demonstrate the advantages of our proposed method as a robust privacy-preserving alternative to the CRE model, we now compare the performances of these two methods in estimating $\boldsymbol{\xi }$, the coefficient for $\boldsymbol{\bar{X}}_i$ in (9). We first simulated a patient-level covariate, $X_{ij}$, from a $\text {N}(m_X,0.25)$ distribution, where $m_X$ was drawn from a $\text {N}(-0.4,0.25)$ distribution. Then, we set $\mu ^*=-6$, $\beta =1$, and $\xi =0.25$ in (9). The random effects, $\gamma ^*_i$, were generated as $\gamma ^*_i=\xi \bar{X}_i+\tau _i$, where $\tau _i$ was simulated from a normal distribution contaminated by outliers. Here, we simulated $Y_{ij}$ from a normal distribution to facilitate later comparisons with robust CRE models for which public software is based on linear mixed models [23, 24].

**Fig. 2:**
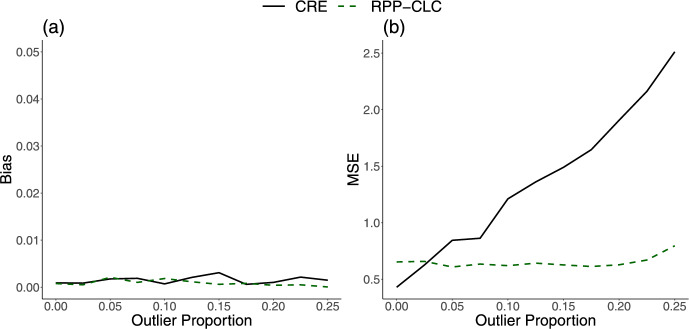
**a** Absolute bias and **b** 100 times the mean squared error (100MSE) for the estimate of $\xi$ (the between-provider confounding effect parameter in the underlying model), from either the Correlated Random Effects (CRE) model or the proposed Robust Privacy-Preserving model for Cluster-Level Confounding (RPP-CLC model). Results are based on 5000 iterations. The true parameter value is $\xi =0.25$, and the proportion of providers with outlying cluster-level treatment effects is varied

As shown in Fig. 2, the estimates from both the CRE model and the RPP-CLC model had low bias for every level of outlier contamination in the random effects. However, the CRE model estimates became much more unstable as outliers were introduced into the random effects distribution, and the MSE increased almost linearly with the outlier proportion. In contrast, the MSE for the RPP-CLC model remained constant, regardless of the outlier proportion (Fig. 2). Several authors have proposed robust versions of the CRE model to improve its precision [20, 22]. While these methods have theoretical validity, they are often computationally intensive for large-scale applications. Table 1 compares the runtimes and memory usages of two popular robust versions of the CRE model [23, 24] and our proposed RPP-CLC model. All models were assessed in 64-bit R software [30]. The robust CRE models were computationally expensive, and even with just 50,000 records, they exhausted the vector memory allocation in R (Table 1), whereas the RPP-CLC model ran quickly with little memory usage.

**Table 1:** Runtime (and memory allocation) comparison among robust Correlated Random Effects (CRE) models and the proposed Robust Privacy-Preserving model for Cluster-Level Confounding (RPP-CLC model). Huberized CRE: robust scoring equations (robustlmm package), Rank CRE: rank-based CRE model (rlme package), RPP-CLC : proposed robust privacy-preserving model

Number of records	10,000	50,000	100,000
Huberized CRE	280.2 s (64.3 GB)	Memory exhausted	Memory exhausted
Rank CRE	5.61 s (8.86 GB)	Memory exhausted	Memory exhausted
RPP-CLC	1.2 s (1.36 GB)	2.09 s (2.38 GB)	4.01 s (2.33 GB)

s *second*, *GB* gigabyte

In our motivating application, national transplant datasets contain millions of records, so we argue that robust patient-level CRE models are unsuitable for such large-scale applications, even if all patient-level data are available. Our RPP-CLC model is much more computationally efficient than the robust CRE models, and it is more stable in the presence of outliers compared to the original CRE model. In addition, if patient-level data are restricted, our method can still be implemented using publicly available summary statistics.

### Inference

Using a similar simulation structure as in the previous section, we explored the properties of our Frequentist and Pseudo-Bayesian “flagging” methods, which are inference methods designed to accurately flag outlying providers with extremely poor quality of care. We considered two different settings for the first provider in our simulated datasets to set flagging targets and highlight the differences in performance across methods. In the first setting, we defined a null provider with $\gamma _1^*=0$ and varied the magnitude of $W_1$. In the second setting, we let $W_1=1$ and varied $\gamma ^*_1$ so that the provider had increasingly low-quality care. For both the naive and adjusted Frequentist methods, we flagged the provider if the Z-score was more extreme than ±1.96, which corresponds to a two-sided test at the 0.05 level. For the Pseudo-Bayesian methods, we flagged the provider if the $95\%$ credible interval did not contain one.

**Fig. 3:**
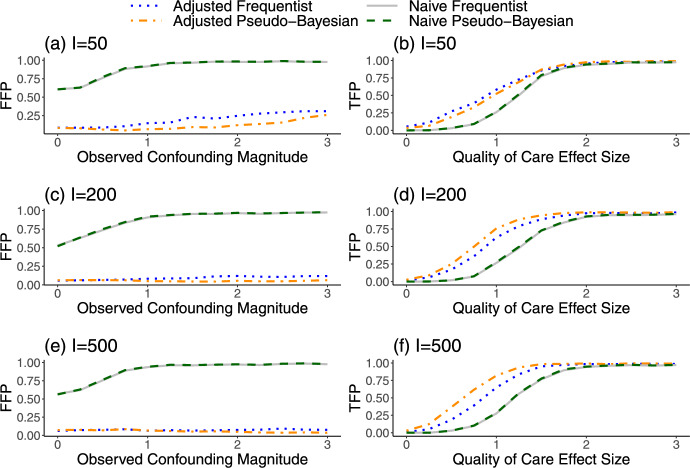
Probability of (**a**, **c**, **e**) falsely flagging a null provider or (**b**, **d**, **f**) correctly flagging a provider with low quality of care, for different levels of confounding or quality of care effect sizes. The Frequentist methods compare standardized Z-scores to an absolute threshold of 1.96. The Pseudo-Bayesian methods compare 95% credible intervals to the null value of one. The naive methods ignore the observed and unobserved provider-level confounding factors, whereas the adjusted versions account for these variables. Results are based on 1000 iterations. *I* number of providers, *FFP* False-Flagging Probability, *TFP* True-Flagging Probability

Figure 3 shows that the “naive” flagging approaches, which ignore $W_i$ and $\alpha _i$, had higher false-flagging probabilities (FFP) and lower true-flagging probabilities (TFP) compared to the adjusted versions. We note that the naive Frequentist approach can also be viewed as a type of conventional federated learning approach because the expected outcome counts that are used to construct the test statistic can be obtained from a federated generalized linear model. However, because the test statistics do not correct for cluster-level confounding, this approach still has major disadvantages compared to our proposed privacy-preserving approach. In Fig. S1 of Online Appendix H, we demonstrate more specifically how the FFPs for the naive method are not only high but also increase rapidly with the magnitude of the unobserved quantity $\alpha _i$, whereas the FFPs for the adjusted method are relatively constant and close to zero across most of the range of $\alpha _i$. This highlights the contributions of the overdispersion correction in reducing sensitivity to $\alpha _i$ during inference.

In general, the naive Frequentist and Pseudo-Bayesian methods produced very similar flagging results, but the adjusted Frequentist approach had a higher FFP relative to the adjusted Pseudo-Bayesian approach. The elevated FFP of the Frequentist approach was much more severe when the number of providers (*I*) was small. These findings are expected, since only the Pseudo-Bayesian approach accounts for uncertainty in $\widehat{\nu }$, and $\widehat{\nu }$ becomes less precise as *I* decreases. In addition, the adjusted Pseudo-Bayesian approach had a higher TFP than the adjusted Frequentist approach for large *I* (i.e., when there is less shrinkage toward the prior mean of zero). We suspect that this improved TFP is a reflection of the fact that the Pseudo-Bayesian approach uses information from the underlying model to incorporate $\alpha _i$ directly into the posterior distribution.

In addition to these flagging properties, we compared the empirical Frequentist coverage probabilities of the credible intervals from our proposed Pseudo-Bayesian approach and the Naive Pseudo-Bayesian approach. Table 2 shows that the credible interval from our proposed method achieves a coverage probability for the true measure value (defined as $\exp (\gamma _i^*)$ under the Poisson simulation model) that is very close to the nominal level of 0.95. In contrast, the credible interval from the Naive Pseudo-Bayesian approach that ignores the impact of $W_i$ and $\alpha _i$ has an extremely low coverage probability.

**Table 2:** Empirical frequentist coverage probabilities of the credible intervals from the naive and adjusted Pseudo-Bayesian inference methods for the true cluster effect parameter $\gamma _i^*$

	Coverage probability	
$\gamma _i^*$	Naive Pseudo-Bayesian	Adjusted Pseudo-Bayesian
0.00	0.04	0.95
0.25	0.04	0.93
0.50	0.04	0.94
0.75	0.05	0.90
1.00	0.05	0.92
1.25	0.06	0.92
1.50	0.05	0.92
1.75	0.08	0.92
2.00	0.09	0.93
2.25	0.11	0.93
2.50	0.09	0.93
2.75	0.12	0.93
3.00	0.14	0.93

The adjusted method takes into account variation from observed and unobserved cluster-level confounding, whereas the naive method ignores these sources of variation

To examine the relative contributions of our corrections for observed cluster-level confounders ($\boldsymbol{W}_i$) and overdispersion from unobserved cluster-level confounders ($\alpha _i$), we repeated our simulation analyses while only correcting for observed confounding and ignoring the potential for overdispersion. In these analyses, we focused on the property of false flagging because inflated false-flagging rates are the main consequence of overdispersion. We show in Fig. S2 that after ignoring overdispersion, the false-flagging rates become highly elevated compared to our original results in Figure 3, though they are still better than entirely naive methods that also ignore the observed cluster-level confounding. These findings highlight that both contributions are important for producing valid inference.

Finally, we performed supplemental simulations that assess the performance of our methods under the alternative null model described in Sect. 3.2.2, which allows $\gamma _i^*$ to be a random quantity with a normal distribution under the null hypothesis. In this simulation, we also set the correlation between $\alpha _i$ and $\gamma _i^*$ for null centers to be 0.71. We observe in Fig. S3 of Online Appendix H that the performance of the adjusted methods was nearly identical to that shown in Figure 3, with controlled false-flagging rates and high true-flagging rates. This result demonstrates that our method is compatible with both of the most commonly used null models in the provider profiling literature [4].

## Kidney Transplant Center Evaluations

### Donor Organ Availability and the COVID-19 Pandemic

Organ Procurement Organizations (OPOs) are independent entities that are responsible for collecting and allocating donor organs to transplant centers within a particular geographic region, referred to as the Donation Service Area (DSA). It has been shown that OPOs vary in their abilities to recover organs at a high rate, and combined with geographic variation in mortality rates and attitudes toward donation, this causes substantial disparities in the availability of donor organs across DSAs [9]. For transplant centers that reside in DSAs with severe shortages in donor organ supply, it is very challenging to meet the needs of their patients and provide transplants quickly, even if these centers deliver high-quality care. Furthermore, several authors have noted that the COVID-19 pandemic has severely impacted the relationships between OPOs and transplant centers, further complicating our efforts to evaluate transplant center performance during and after the pandemic years [8, 10, 31]. Thus, in order to develop fair transplant center evaluations, we must consider these cluster-level confounding factors in the analysis. While data on donor organ availability across DSAs can easily be obtained from the SRTR’s public reports, it is difficult to fully describe pandemic-related changes in organ allocation efficiency with observable variables.

**Fig. 4:**
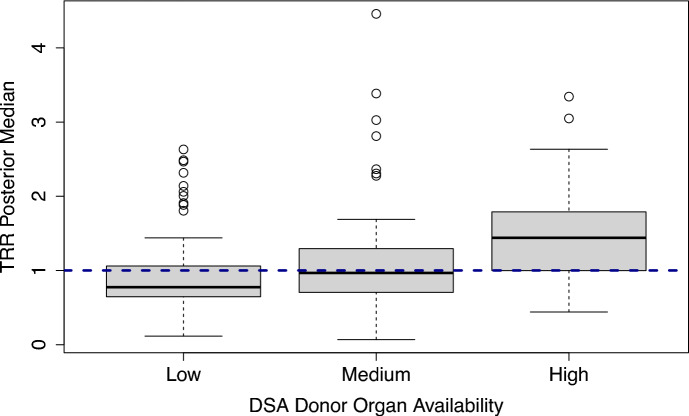
Distribution of the posterior medians for the Transplant Rate Ratio (TRR) measure, stratified by donor organ availability within the centers’ Donation Service Areas (DSAs). The posterior median for each transplant center is computed from the SRTR’s proposed Gamma distribution, which does not account for geographic variation in donor organ availability. Donor organ availability is defined as the number of donors per transplant candidate within a center’s DSA (“Low,” “Medium,” and “High” groups are formed by tertiles). The horizontal dashed line is the reference line of TRR = 1, which indicates that a center is consistent with national norms, and higher TRR values indicate better performance in terms of access to transplantation. Centers that reside in DSAs with high donor organ availability tend to have better evaluations based on the SRTR’s metrics

Currently, the SRTR’s transplant center quality metrics are only adjusted for patient-level risk factors, and the corresponding inference approaches do not control for cluster-level confounders such as geographic disparities in donor organ availability or COVID-19 effects. For example, the SRTR’s Transplant Rate Ratio (TRR) measure describes a center’s observed number of transplants relative to the expected number under the national average, adjusted only for between-center variation in patient characteristics such as demographic and clinical information [1]. Figure 4 illuminates the consequences of ignoring cluster-level confounding by showing the distributions of the SRTR’s posterior medians for the TRR measure, stratified by the regional supply of donor organs. These descriptive plots suggest that centers with larger supplies of donor organs tend to be evaluated much more favorably than those with fewer resources, and the SRTR’s current evaluation system may in large part reflect the DSAs’ donor organ supplies, as opposed to the transplant centers’ true healthcare quality. To address this gap, we use summary statistics from the SRTR’s public reports and our proposed methods to evaluate transplant center quality, controlling for the availability of donor organs within the centers’ DSAs and for overdispersion due to residual unobserved confounding, such as the COVID-19 effects that are difficult to measure. In this way, we aim to make the evaluations more fair for centers with low donor organ supplies or severe pandemic-related disruptions in their organ allocation network. Through our privacy-preserving analysis of the TRR values, we are able to assess transplant center quality without ever having to fit the patient-level model in (1).

### SRTR Data

We collected center-level and DSA-level summary statistics from the January 2022 public releases of the SRTR’s transplant program and OPO-specific reports [1]. The observed and expected numbers of transplants for the centers were based on a 2-year cohort of waitlisted patients from July 1, 2019 to June 30, 2021. The DSA-level counts of donors and waitlisted patients, which we used to define our adjustment variable, were based on a one-year cohort from July 1, 2020 to June 30, 2021. Thus, the data were measured during the height of the pandemic. We defined the observed cluster-level adjustment variable, $W_i$, as the number of donors (meeting eligibility criteria) per patient on the transplant waitlist within the *i*th center’s DSA. To implement the model proposed in Sect. 3.2, we first calculated the naive fixed-effects Z-scores from the public summary data as follows:

$$\begin{aligned} Z_{FE,i}=\frac{\text {Observed Number of Transplants-Expected Number of Transplants}}{\sqrt{\text {Expected Number of Transplants}}}. \end{aligned}$$

Then, we defined the conditional mean of $Z_{FE,i}$ in terms of the observed cluster-level confounder (i.e., DSA donation rate) according to Eq. (6) as follows:

$$\begin{aligned} E[Z_{FE,i};\gamma _i^*=0]=\sqrt{\tilde{n}_i}(\exp (\nu \{\text {DSA Donation Rate}\}+\sigma ^2_{\alpha }/2)-1 ), \end{aligned}$$

and we defined the conditional variance similarly by applying Eq. (6). From this model specification, we robustly estimated the cluster-level confounding parameters $\nu$ and $\sigma ^2_{\alpha }$ based on the likelihood function in Eq. (8), and we used these estimates to calculate the posterior distributions of the TRRs for inference according to Eq. (11).

### Analysis Results

**Fig. 5:**
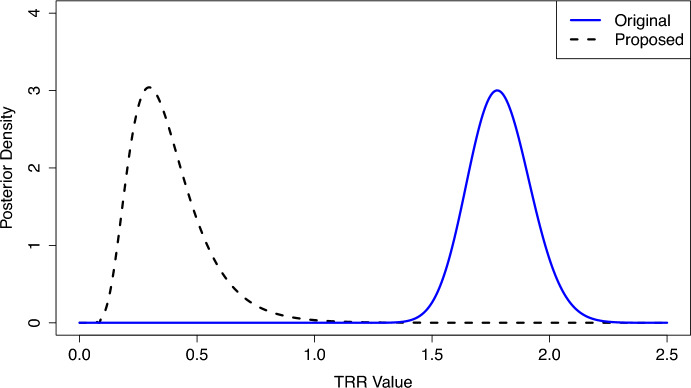
Original and proposed posterior distributions for the Transplant Rate Ratio (TRR) of one U.S. kidney transplant center. The proposed posterior distribution is adjusted for geographic disparities in donor organ availability and unobserved confounding factors, whereas the original posterior distribution is not adjusted for these factors. A TRR value of one indicates that the provider is consistent with the national norms, and lower TRR values indicate worse performance

We found that $W_i$ had a strong confounding effect on the TRR values. From our proposed estimation procedure, $\widehat{\nu }=4.47$ with a 95% confidence interval of (3.40, 5.55). The example center in Fig. 5 resides in a DSA with very high donor organ availability, and while this center appeared to be performing transplants quickly according to the SRTR’s original posterior distribution, $f_{R_i|O_i,E_i}(r_i)$, the results based on our proposed posterior distribution, $f_{R^*_i|O_i,E_i}(r^*_i)$, suggest that this center should be transplanting patients at a higher rate given the ample supply of donor organs in its DSA. Among the centers that were flagged as poor performers based on the credible intervals of $f_{R_i|O_i,E_i}(r_i)$, 79% were reclassified as null centers based on the credible intervals of $f_{R^*_i|O_i,E_i}(r^*_i)$, which adjusts for $W_i$ and overdispersion from $\alpha _i$. From the results in Sect. 4, we suspect that many of these centers were falsely flagged originally. We also identified seven new centers as poor performers after adjusting for the confounding effects.

**Fig. 6:**
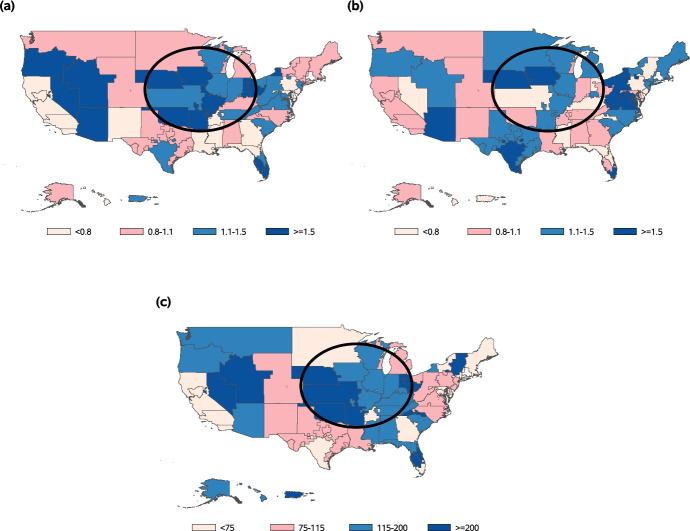
Maps of average Transplant Rate Ratio (TRR) posterior medians and the provider-level adjustment variable ($W_i$), by Donation Service Area (DSA). An example region with high donor organ availability is circled. **a** Average TRR posterior median among centers in the DSA, not adjusting for $W_i$, **b** average TRR posterior median among centers in the DSA, adjusting for $W_i$, **c** $W_i$: Number of donors per 1000 transplant candidates within the DSA

The DSA-level map in Fig. 6a shows that the transplant centers with the best evaluations, based on the SRTR’s original posterior distribution, were concentrated in certain areas of the U.S. (e.g., the circled region in Fig. 6). These regions also had some of the highest levels of donor organ availability in the nation (Fig. 6c). After adjusting for $W_i$ and correcting for overdispersion from $\alpha _i$, using our proposed methods, this spatial correlation became less apparent (Figs. 6b, c), providing evidence that our adjustment appropriately controls for the geographic confounding effects. We note that EN inference methods from the existing literature [15, 16] would not be able to adjust for the geographic disparities in donor organ availability (i.e., the observed cluster-level confounding variable) in this application, losing valuable information that is highly relevant to transplant center performance.

As in Sect. 4, we assess the relative contributions of our corrections for observed cluster-level confounding and unobserved cluster-level heterogeneity by recreating Figure 5 with an additional posterior distribution that only corrects for $\boldsymbol{W}_i$ and not the variability in $\alpha _i$. As expected, we find in Fig. S4 of Online Appendix H that the posterior distribution which ignores the influence of $\alpha _i$ is much narrower than the distribution from our proposed method, overstating the amount of certainty in the underlying TRR value. Thus, these analyses highlight again that both corrections are essential to achieve valid inference.

## Discussion

We have developed a privacy-preserving framework to estimate and adjust for the confounding effects of observable cluster-level risk factors and overdispersion from unobservable factors when identifying transplant centers with significant treatment effects. The proposed confounding effect estimator is highly robust to outlying clusters and only depends on summary information that is widely used in the transplantation field. The proposed Pseudo-Bayesian inference procedure can incorporate these estimates while recognizing their statistical uncertainty and correcting for the inevitable influence of unobserved confounding. These aspects of our proposed method are major advantages over existing EN corrections, which completely lack the ability to adjust for observable cluster-level confounding factors.

Most privacy-preserving models rely on distributed optimization algorithms, where each organization repeatedly calculates a set of statistics through multiple communications [11, 12]. In this paper, we have shown that one may estimate cluster-level confounding effects simply by downloading publicly available summary statistics from the SRTR and performing a single model-fitting routine. This provides a convenient alternative for stakeholders to refine provider evaluations, without any organization needing to share or reanalyze patient-level data. Even in applications where the full patient-level data are available, the proposed RPP-CLC model may be much more computationally efficient than robust CRE models, as we showed in Sect. 4.

Privacy-preservation, outlier-robust estimation, and computational efficiency are important goals in the analysis of clustered data across a wide range of applications. While our models are motivated by these issues in the context of transplant center evaluation, the proposed solutions are also useful in more general settings where any of these challenges arise. Similarly, our inference methods are motivated by the detection of transplant centers that deliver extremely low-quality care, but they also address more general issues in cluster-level outlier detection including adjustment of observed cluster-level confounders and overdispersion due to unobserved confounding, which are common in observational studies.

In many applications, the cluster size $n_i$ may be informative for the underlying cluster effect $\gamma _i^*$. For example, in healthcare quality evaluations, larger providers may tend to have more resources to provide better treatment. Under these scenarios, an important practical consideration is whether to adjust for cluster size or not in the proposed model. If the goal is to evaluate clusters relative to other clusters of similar size, then it may be advantageous to adjust for cluster size in the model as an element of $\boldsymbol{W}_i$. However, if the goal is to identify outlying providers relative to the national average, regardless of whether they are large or small, then we advise against adjusting for cluster size in the model. Future work may explore the statistical properties of empirical null methods under this scenario. Furthermore, many provider profiling applications are focused on evaluating healthcare facilities such as hospitals that treat a large number of patients, where it is reasonable to assume that the asymptotic properties of the Z-score statistics hold. However, other applications may be focused on individual practitioners or other clustering units with small sample sizes. We have extended empirical null methods to these settings with small cluster sizes in a separate publication through exact inference methods, and these approaches fit within the general framework of the methods proposed in this paper [32].

Cluster-level confounding (whether observed or unobserved) may come from inherent geographic differences in factors that are related to the physical locations of the clusters of interest. When these factors are measurable, it is possible to adjust for them through $\boldsymbol{W}_i$ as demonstrated in our motivating application in Sect. 5. However, future work may extend the proposed methods to address overdispersion from latent geographic variation in the unobservable $\alpha _i$ while accounting for spatial autocorrelation. In this way, individualized empirical null methods that are fully integrated with spatial modeling would further expand the potential of cluster-level confounding models.

Our proposed methodology adopts a general inference framework that is commonly used in healthcare provider profiling, where the objective is to identify outlying providers with truly extreme deviations from the national norm, and it is assumed that almost all between-provider variation in patient outcomes is due to clinically meaningless fluctuations in healthcare quality or unobserved confounding. [15] consider an alternative model that attempts to only account for the fraction of between-provider variation that comes from unobserved confounding, as opposed to fluctuation in healthcare quality. However, they note that the data do not contain any information to identify this true fraction, so it must be specified based on assumptions or expert opinions about the performance of the risk-adjustment model. Regardless, this approach can be implemented along with our proposed methods in applications by taking a fraction of the $\sigma ^2_{\alpha }$ term in all inference procedures.

Through our real data analyses, we have found that adjusting for geographic disparities in donor organ availability and unobservable confounding factors, using our proposed methods, can substantially change the interpretations of U.S. transplant center performance. These new analyses are especially useful for efforts to expand access to transplantation and to find the sources of inequitable care. With our novel robust privacy-preserving model and inference procedure, researchers can conveniently and efficiently perform accurate assessments to improve the quality and equity of transplant care.

## Supplementary Information

Below is the link to the electronic supplementary material.

Supplementary file 1 (pdf 298 KB)

## Figures and Tables

**Algorithm 1:**
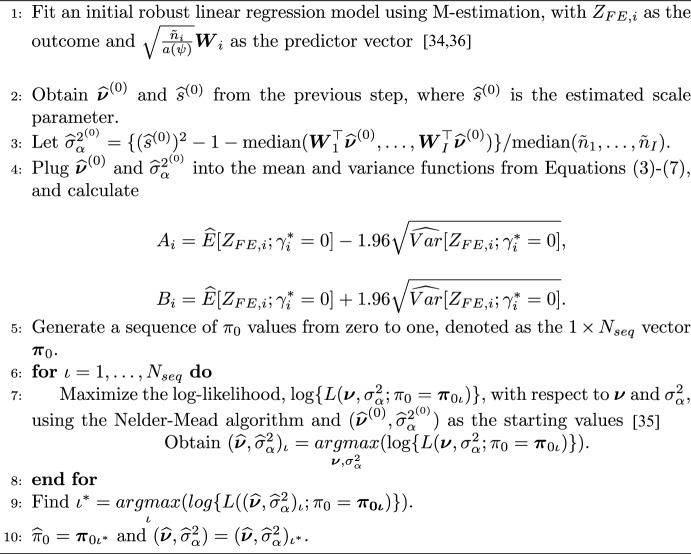
Model fitting algorithm [34–36]

## Data Availability

The data that support the findings of this paper are publicly available from the SRTR website (https://www.srtr.org/about-the-data/technical-methods-for-the-program-specific-reports/).

## Appendix 1: Model Form

Assume that all expectations are conditional on $\sum _{j=1}^{n_i} b'(\theta ^0_{ij})$, $\tilde{n}_i=\sum _{j=1}^{n_i} b''(\theta ^0_{ij})$, and $\boldsymbol{W}_i$. The conditional null mean of the summary-level naive Z-score is

$$E[Z_{FE,i};\gamma ^*_i=0]=\frac{1}{\sqrt{\tilde{n}_ia(\psi )}}E\left[ \sum _{j=1}^{n_i} b'(\theta _{ij}^*)-\sum _{j=1}^{n_i} b'(\theta _{ij}^0) ; \gamma _i^*=0\right] .$$

Extending the first-order Taylor series expansions described in [16] to our setting with both observed and unobserved cluster-level confounding (i.e., $b'(\theta ^*_{ij})$ and $b''(\theta ^*_{ij})$ around $\theta ^0_{ij}$),

$$\begin{aligned} E[Z_{FE,i};\gamma ^*_i=0]&\approx \frac{1}{\sqrt{\tilde{n}_ia(\psi )}} E\left[ \tilde{n}_i(\theta ^*_{ij}-\theta ^0_{ij}) ;\gamma _i^*=0\right] \\&=\sqrt{\frac{\tilde{n}_i}{a(\psi )}}E[\boldsymbol{W}_i^{\top }\boldsymbol{\nu }+\alpha _i] \\&=\sqrt{\frac{\tilde{n}_i}{a(\psi )}}\boldsymbol{W}_i^{\top } \boldsymbol{\nu } \hspace{25pt} (\text {if } \hspace{6pt} E[\alpha _i|\boldsymbol{W}_i,\boldsymbol{X}_{i1},\ldots ,\boldsymbol{X}_{in_i}]=0). \\ \end{aligned}$$

If $\gamma ^*_i$ is random and $E[\gamma ^*_i|\boldsymbol{W}_i,\boldsymbol{X}_{i1},\ldots ,\boldsymbol{X}_{in_i}]=0$, then we can replace $\alpha _i$ above with $\gamma _i=\alpha _i+\gamma ^*_i$, and the result still holds. $Var[Z_{FE,i};\gamma ^*_i=0]$ is derived in a similar manner as above, utilizing a similar variance decomposition and Taylor series approximation as in [16]. For any outcome distribution in the exponential family,

$$\begin{aligned} Var[Z_{FE,i};\gamma ^*_i=0]&\approx \frac{1}{\tilde{n}_ia(\psi )}Var[\tilde{n}_i(\boldsymbol{W}_i^{\top }\boldsymbol{\nu }+\alpha _i)] \\&+ E\left[ 1+\frac{\sum _{j=1}^{n_i}b'''(\theta ^0_{ij})}{\tilde{n}_i}(\boldsymbol{W}_i^{\top }\boldsymbol{\nu }+\alpha _i )\right] \\&=1+\frac{\sum _{j=1}^{n_i}b'''(\theta ^0_{ij})}{\tilde{n}_i} \boldsymbol{W}_i^{\top } \boldsymbol{\nu }+\varphi \tilde{n}_i, \end{aligned}$$

where $$\varphi =\sigma ^2_{\alpha }/a(\psi )$$.

## Appendix 2: Normal Distribution Special Case

When $Y_{ij}$ follows a normal distribution,

$$\begin{aligned} E[Z_{FE,i};\gamma ^*_i=0]&=E[E[Z_{FE,i}|\alpha _i,\gamma ^*_i=0];\gamma ^*_i=0] \nonumber \\&=\frac{1}{\sqrt{n_i\sigma ^2_{\varepsilon }}}E\left[ \sum _{j=1}^{n_i} b'(\theta _{ij}^*)-\sum _{j=1}^{n_i} b'(\theta _{ij}^0);\gamma _i^*=0\right] \\&=\frac{1}{\sqrt{n_i\sigma ^2_{\varepsilon }}}E\left[ \sum _{j=1}^{n_i} \theta _{ij}^*-\sum _{j=1}^{n_i} \theta _{ij}^0;\gamma _i^*=0\right] \\&=\sqrt{\frac{n_i}{\sigma ^2_{\varepsilon }}}\boldsymbol{W}_i^{\top }\boldsymbol{\nu } \hspace{25pt} (\text {if} \hspace{6pt} E[\alpha _i|\boldsymbol{W}_i,\boldsymbol{X}_{i1},\ldots ,\boldsymbol{X}_{in_i}]=0). \end{aligned}$$

Similarly,

$$\begin{aligned} Var[Z_{FE,i};\gamma ^*_i=0]&=Var[E[Z_{FE,i}|\alpha _i,\gamma ^*_i=0];\gamma ^*_i=0]\\ &\quad +E[Var[Z_{FE,i}|\alpha _i,\gamma ^*_i=0];\gamma ^*_i=0] \\&=\frac{1}{n_i \sigma ^2_{\varepsilon }} Var[n_i(\boldsymbol{W}_i^{\top }\boldsymbol{\nu }+\alpha _i);\gamma _i^*=0] \\ &\quad +\frac{1}{n_i\sigma _{\varepsilon }^2}E\left[ Var\left[ \sum _{j=1}^{n_i}Y_{ij}\right] ;\gamma _i^*=0\right] \\&=1+\frac{\sigma ^2_{\alpha }}{\sigma ^2_{\varepsilon }} n_i. \end{aligned}$$

## Appendix 3: Poisson Distribution Special Case

Under the Poisson model, $b(\cdot )=\exp (\cdot )$ and $a(\psi )=1$. Since $\alpha _i \sim \text {N}(0,\sigma _{\alpha }^2$), $\exp (\alpha _i) \sim \text {Lognormal}(0,\sigma ^2_{\alpha }$). The null mean and variance functions follow directly from this result, combined with the approach in Appendix 1. These arguments also apply to the Quasi-Poisson model, with $a(\psi )=\psi$, where $\psi$ may be different from one.

## Appendix 4: Model Estimation Algorithm

Our proposed model-fitting algorithm is summarized below (extended from [16] to accommodate both observed and unobserved cluster-level confounding):

## Appendix 5: Theoretical Connections With the CRE Model

Under the CRE assumptions, $\gamma _i^*=\boldsymbol{\bar{X}}_i^{\top }\boldsymbol{\xi }+\tau _i$, where $\tau _i|\boldsymbol{X}_{i1},\ldots ,\boldsymbol{X}_{in_i} \sim N(0,\sigma ^2_{\tau })$. Therefore, $E[\tau _i|\boldsymbol{X}_{i1},\ldots ,\boldsymbol{X}_{in_i}]=0$ and $\theta ^*_{ij}-\theta ^0_{ij}=\tau _i+\boldsymbol{\bar{X}}_i^{\top }\boldsymbol{\xi }$. It follows directly from the arguments of Appendix 1 (substituting $\tau _i$ for $\alpha _i$ and $\boldsymbol{\bar{X}}_i$ for $\boldsymbol{W}_i$) that

$$E[Z_{FE,i};\gamma ^*_i=0]\approx \sqrt{\frac{\tilde{n}_i}{a(\psi )}}\boldsymbol{\bar{X}}_i^{\top }\boldsymbol{\xi }.$$

The exact mean and variance functions for the normal and Poisson models follow directly from the arguments above, combined with those from Appendices 2 and 3.

## Appendix 6: Variance of the Confounding Effect Estimate

Let $\boldsymbol{W^*}$ be an $I_0$ by *P* matrix with *i*th row $\sqrt{\frac{\tilde{n}_i}{a(\psi )}}\boldsymbol{W}_i$, where *P* is the number of observed cluster-level confounding variables, and let $\boldsymbol{W^*}_{\text {null}}$ be the submatrix that corresponds to null providers. Since we do not know exactly which providers are null, we obtain $\boldsymbol{W^*}_{\text {null}}$ by selecting all rows of $\boldsymbol{W^*}$ that correspond to $Z_{FE,i}$ values falling within the assumed null intervals $[A_i, B_i]$ from Sect. 3.2. From Eqs. (3) and (4), $Z_{FE,i}$ approximately follows a linear model with heteroskedastic errors for all centers included in $\boldsymbol{W^*}_{\text {null}}$. Applying the covariance estimator formula from [33], $\boldsymbol{\Sigma _{\widehat{\nu }}}$ is consistently estimated by

$$(\boldsymbol{W^*_{\text {null}}}^{\top }\boldsymbol{W^*_{\text {null}}})^{-1}\boldsymbol{W^*_{\text {null}}}^{\top }\boldsymbol{\Omega W^*_{\text {null}}}(\boldsymbol{W^*_{\text {null}}}^{\top }\boldsymbol{W^*_{\text {null}}})^{-1}$$

where $\boldsymbol{\Omega }$ is an $I_0$ by $I_0$ matrix with diagonal elements $Var[Z_{FE,i}|\boldsymbol{W}_i,\gamma _i^*=0]$ and off-diagonal elements of zero. The posterior distribution for the parameter of interest can be approximated by plugging in a consistent estimator for the nuisance parameter $\boldsymbol{\Sigma }_{\boldsymbol{\widehat{\nu }}}$.

## Appendix 7: Pseudo-Bayesian Inference Approach

Under the SRTR’s Gamma-Poisson model, it is assumed that $O_i|E_i \sim \text {Poisson}(R_i E_i)$, where $R_i \sim \text {Gamma}(2,2)$. This yields the SRTR’s posterior distribution, denoted as $f_{R_i|O_i,E_i}(r_i)$:

$$R_i | O_i, E_i \sim \text {Gamma}(O_i+2,E_i+2).$$

Under our proposed model, $O_i|E_i,\Lambda _i \sim \text {Poisson}(R_i^* E_i \lambda _i)$, where $R_i^* \sim \text {Gamma}(2,2)$. This yields the following conditional posterior distribution ($f_{R_i^*|O_i,E_i,\Lambda _i}(r_i^*)$):

$$R_i^* | O_i, E_i, \Lambda _i \sim \text {Gamma}(O_i+2,E_i\lambda _i+2).$$

After defining the distribution of $\Lambda _i|O_i,E_i$, which we denote as $f_{\Lambda _i|O_i,E_i}(\lambda _i)$, we apply the Law of Total Probability to derive the distribution of $R_i^*|O_i,E_i$:

$$f_{R^*_i|O_i,E_i}(r^*_i)=\int f_{R_i^*|O_i,E_i,\Lambda _i}(r^*_i)f_{\Lambda _i|O_i,E_i}(\lambda _i) \mathrm{d}\lambda _i.$$

## Code Availability

The corresponding R codes are available at https://github.com/nhart985/RPPCLC.

## References

[1] Scientific Registry of Transplant Recipients (2022) Technical methods for the program specific reports. https://www.srtr.org/about-the-data/technical -methods-for-the-program-specific-reports/

[2] Howard R, Cornell D, Schold J (2009) CMS oversight, OPOs and transplant centers and the law of unintended consequences. Clin Transplant 23(6):778–783. 10.1111/j.1399-0012.2009.01157.x 20447183 10.1111/j.1399-0012.2009.01157.x

[3] Jay C, Schold JD (2017) Measuring transplant center performance: the goals are not controversial but the methods and consequences can be. Curr Transplant Rep 4(1):52–58. 10.1007/s40472-017-01389 28966901 10.1007/s40472-017-0138-9 PMC5616160

[4] Jones H, Spiegelhalter D (2011) The identification of unusual health-care providers from a hierarchical model. Am Stat 65(3):154–163. 10.1198/tast.2011.10190

[5] He K, Kalbfleisch J, Li Y, Li Y (2013) Evaluating hospital readmission rates in dialysis facilities; adjusting for hospital effects. Lifetime Data Anal 19(4):490–512. 10.1007/s10985-013-9264-6 23709309 10.1007/s10985-013-9264-6

[6] Chen B, Lawson KA, Finelli A, Saarela O (2020) Causal variance decompositions for institutional comparisons in healthcare. Stat Methods Med Res 29(7):1972–1986. 10.1177/0962280219880571 31603028 10.1177/0962280219880571

[7] Tang TS, Austin PC, Lawson KA, Finelli A, Saarela O (2020) Constructing inverse probability weights for institutional comparisons in healthcare. Stat Med 39(23):3156–3172. 10.1002/sim.8657 32578909 10.1002/sim.8657

[8] Miller J, Wey A, Musgrove D, Son Ahn Y, Hart A, Kasiske B, Snyder J (2021) Mortality among solid organ waitlist candidates during COVID-19 in the United States. Am J Transplant 21(6):2262–2268. 10.1111/ajt.16550 33621421 10.1111/ajt.16550 PMC8014331

[9] King K, Husain S, Mohan S (2019) Geographic variation in the availability of deceased donor kidneys per wait-listed candidate in the United States. Kidney Int Rep 4(11):1630–1633. 10.1016/j.ekir.2019.0834.018 31891004 10.1016/j.ekir.2019.08.018 PMC6933455

[10] Hudgins J, Boyer A, Orr K, Hostetler C, Orlowski J, Squires R (2021) The impact and implications of the COVID-19 pandemic on organ procurement outside of an epicenter. Prog Transplant 31(2):171–173. 10.1177/15269248211002808 33722146 10.1177/15269248211002808

[11] Jochems A, Deist TM, van Soest J, Eble M, Bulens P, Coucke P et al (2016) Distributed learning: developing a predictive model based on data from multiple hospitals without data leaving the hospital–a real life proof of concept. Radiother Oncol 121(3):459–467. 10.1016/j.radonc.2016.10.002 28029405 10.1016/j.radonc.2016.10.002

[12] Duan R, Boland M, Liu Z, Liu Y, Chang H, Xu H, Chen Y (2020) Learning from electronic health records across multiple sites: a communication efficient and privacy-preserving distributed algorithm. J Am Med Inform Assoc 27(3):376–385. 10.1093/jamia/ocz199 31816040 10.1093/jamia/ocz199 PMC7025371

[13] Han L, Li Y, Niknam B, Zubizarreta J (2022) Privacy-preserving and communication-efficient causal inference for hospital quality measurement. arXiv:2203.00768

[14] Spiegelhalter D (2005) Funnel plots for comparing institutional performance. Stat Med 24(8):1185–1202. 10.1002/sim.1970 15568194 10.1002/sim.1970

[15] Xia L, He K, Li Y, Kalbfleisch J (2022) Accounting for total variation and robustness in profiling health care providers. Biostatistics 23(1):257–273. 10.1093/biostatistics/kxaa024 32530460 10.1093/biostatistics/kxaa024

[16] Hartman N, Messana J, Kang J, Naik A, Shearon T, He K (2023) Composite scores for transplant center evaluation: a new individualized empirical null method. Ann Appl Stat 18(1):729–748. 10.1214/23-aoas1809 10.1214/23-aoas1809 PMC11395314 39281709

[17] Kalbfleisch J, Wolfe R (2013) On monitoring outcomes of medical providers. Stat Biosci 5(2):286–302. 10.1007/s12561-013-9093-x

[18] Neuhaus J, Kalbfleisch J (1998) Between- and within-cluster covariate effects in the analysis of clustered data. Biometrics 54(2):638–645. 10.2307/3109770 9629647

[19] Wooldridge J (2010) Econometric analysis of cross section and panel data, 2nd edn. MIT, Cambridge

[20] Pinheiro J, Liu C, Wu Y (2001) Efficient algorithms for robust estimation in linear mixed-effects models using the multivariate t distribution. J Comput Graph Stat 10(2):249–276. 10.1198/10618600152628059

[21] Schielzeth H, Dingemanse N, Nakagawa S, Westneat D, Allegue H, Teplitsky C, Sutherland C (2020) Robustness of linear mixed-effects models to violations of distributional assumptions. Methods Ecol Evol 11(9):1141–1152. 10.1111/2041-210X.13434

[22] Kloke J, McKean J, Rashid M (2009) Rank-based estimation and associated inferences for linear models with cluster correlated errors. J Am Stat Assoc 104(485):384–390. 10.1198/jasa.2009.0116

[23] Koller M (2016) robustlmm: an R package for robust estimation of linear mixed effects models. J Stat Softw 75(6):1–24. 10.18637/jss.v075.i06 10.18637/jss.v075.i01 PMC7351245 32655332

[24] Bilgic Y, Susmann H, McKean J (2018) rlme: rank-based estimation and prediction in random effects nested models. R package version 0.5. https://CRAN.R-project.org/package=rlme

[25] Kalbfleisch J, He K, Xia L, Li Y (2018) Does the inter-unit reliability (IUR) measure reliability? Health Serv Outcomes Res Method 18(3):215–225. 10.1007/s10742-018-0185-4

[26] Efron B (2004) Large-scale simultaneous hypothesis testing: the choice of a null hypothesis. J Am Stat Assoc 99(465):96–104. 10.1198/016214504000000089

[27] Efron B (2007) Size, power and false discovery rates. Ann Stat 35(4):1351–1377. 10.1214/009053606000001460

[28] Spiegelhalter D, Sherlaw-Johnson C, Bardsley M, Blunt I, Wood C, Grigg O (2012) Statistical methods for healthcare regulation: rating, screening and surveillance. J R Stat Soc 175(1):1–47. 10.1111/j.1467-985X.2011.01010.x

[29] Salkowski N, Snyder J, Zaun D, Leighton T, Israni A, Kasiske B (2014) Bayesian methods for assessing transplant program performance. Am J Transplant 14(6):1271–1276. 10.1111/ajt.12707 24787026 10.1111/ajt.12707

[30] R Core Team (2021) R: a language and environment for statistical computing. R Core Team, Vienna. https://www.R-project.org/

[31] Bloom Works (2022) The costly effects of an outdated organ donation system: COVID-19 impact on organs. https://bloomworks.digital/organdonationreform/COVID-19/

[32] Hartman N, He K (2024) Individualized empirical null estimation for exact tests of healthcare quality. Stat Med 43(12):2403–2420. 10.1002/sim.10074 38590087 10.1002/sim.10074 PMC11698226

[33] White H (1980) A heteroskedasticity-consistent covariance matrix estimator and a direct test for heteroskedasticity. Econometrica 48(4):817–838. 10.2307/1912934

[34] Huber P (1981) Robust statistics. Wiley, New York

[35] Nelder J, Mead R (1965) A simplex method for function minimization. Comput J 7(4):308–313. 10.1093/comjnl/7.4.308

[36] Venables WN, Ripley BD (2002) Modern applied statistics with S, 4th edn. Springer, New York

