# Supplementary material for: Robust Privacy-Preserving Models for Cluster-Level Confounding: Recognizing Disparities in Access to Transplantation
Source: Stat Biosci. 2025 Jul 7;18(2):341–71. doi: 10.1007/s12561-025-09496-3 (PMC12830051; doi:10.1007/s12561-025-09496-3)
Supplement: Supplementary file 1 — (pdf 298 KB) [file 12561_2025_9496_MOESM1_ESM.pdf]

Appendix H: Supplemental Figures

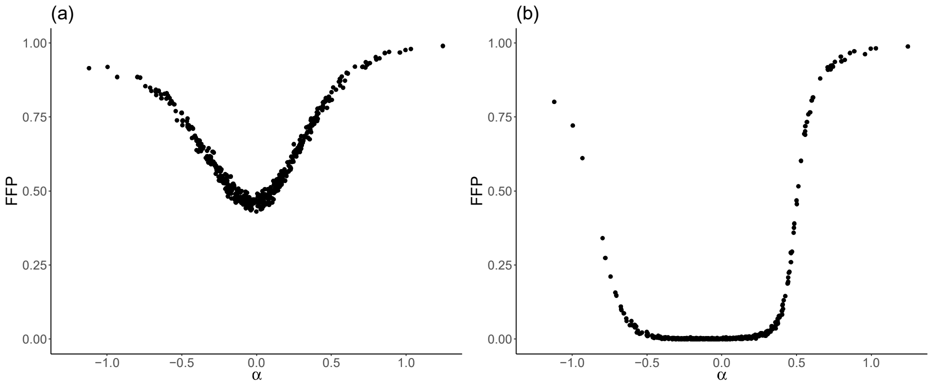

Fig. S1 Probability of falsely flagging a null provider based on (a) the naive Frequentist method that does not account for confounding or (b) the proposed Frequentist method that adjusts for observed cluster-level confounding and overdispersion from the unobserved quantity α_i. Results are based on 1000 iterations, with $I = 500$ providers. FFP: False-Flagging Probability.

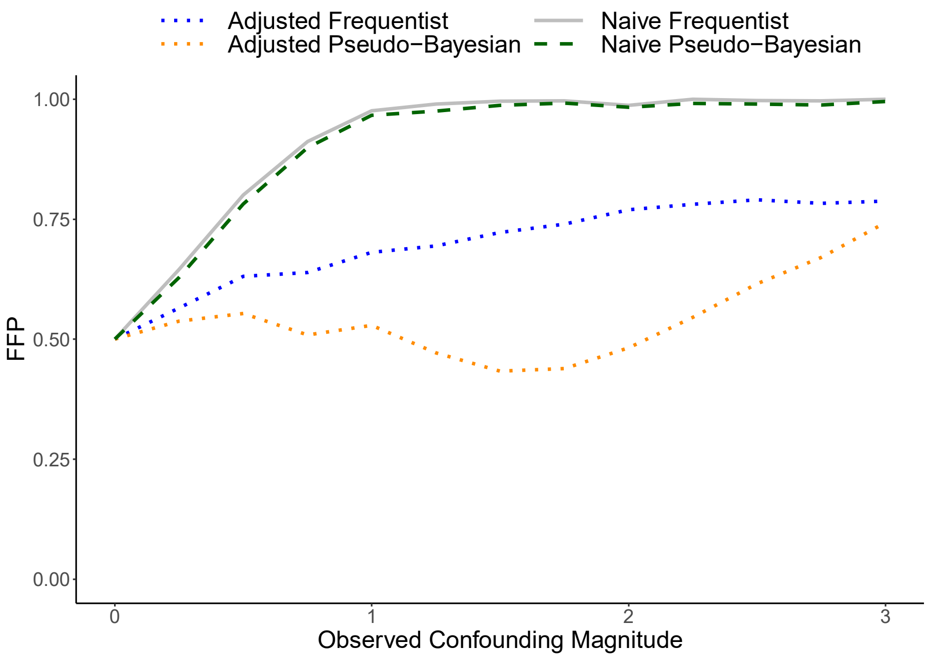

Fig. S2 Probability of falsely flagging a null provider for different levels of observed confounding. The Frequentist methods compare standardized Z-scores to an absolute threshold of 1.96. The Pseudo-Bayesian methods compare 95% credible intervals to the null value of one. The naive methods ignore the observed and unobserved provider-level confounding factors, and the adjusted versions in this simulation only account for the observed confounders. Results are based on 1000 iterations, with $I = 500$ providers. FFP: False-Flagging Probability.

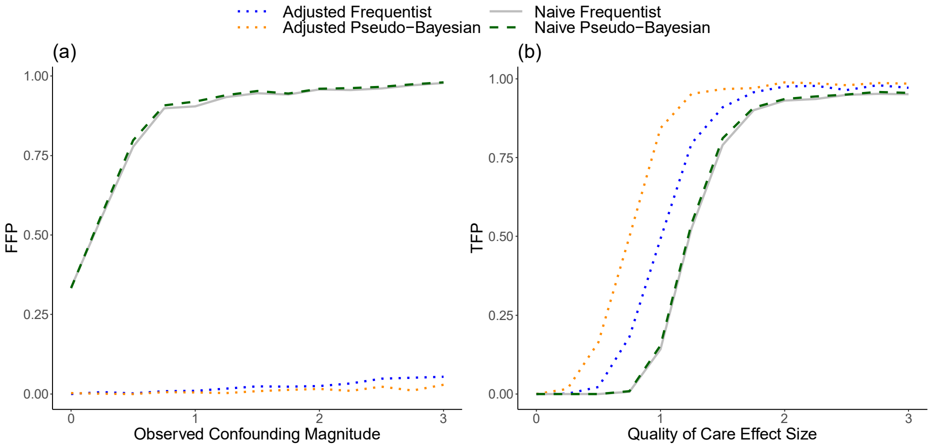

Fig. S3 Probability of (a) falsely flagging a null provider or (b) correctly flagging a provider with low quality of care, for different levels of confounding or quality of care effect sizes and based on the alternative null model (with random γ_i^*). The Frequentist methods compare standardized Z-scores to an absolute threshold of 1.96. The Pseudo-Bayesian methods compare 95% credible intervals to the null value of one. The naive methods ignore the observed and unobserved provider-level confounding factors, whereas the adjusted versions account for these variables. Results are based on 1000 iterations, with $I = 500$ providers. FFP: False-Flagging Probability, TFP: True-Flagging Probability

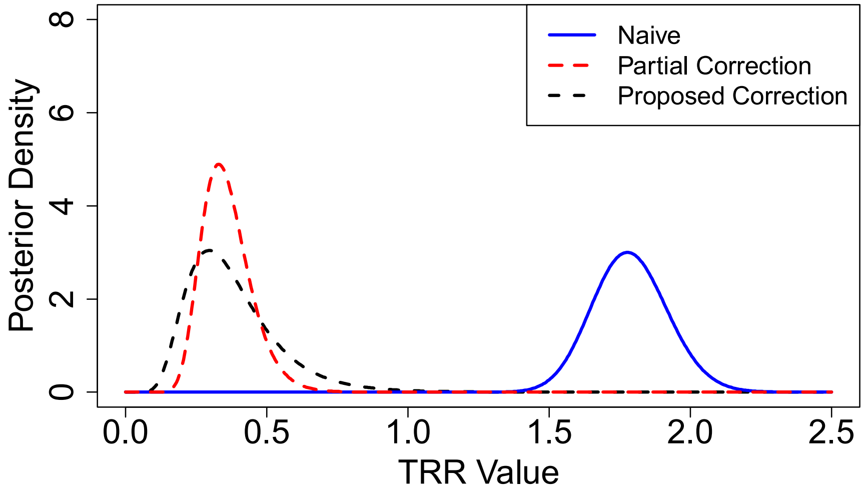

Fig. S4 Posterior distributions for the Transplant Rate Ratio (TRR) of one U.S. kidney transplant center, using different inference methods. The naive approach is based on the SRTR's original method, which does not adjust for observed geographic disparities in donor organ availability or correct for residual unobserved heterogeneity. The partial correction only adjusts for geographic disparities in donor organ availability, and the proposed correction accounts for both observed geographic disparities in donor organ availability and residual unobserved heterogeneity. A TRR value of one indicates that the provider is consistent with the national norms, and lower TRR values indicate worse performance.
